# 1-(2-Bromobenzoyl)-6,7-(methylene­dioxy)isoquinoline

**DOI:** 10.1107/S1600536810051329

**Published:** 2010-12-11

**Authors:** Supakit Achiwawanich, Nisachon Khunnawutmanotham, Supanna Techasakul, Narongsuk Chaichit, Sutatip Siripaisarnpipat

**Affiliations:** a Center of Excellence in Functional Materials, Department of Chemistry, Faculty of Science, Kasetsart University, Bangkok 10903, Thailand; b Chulabhorn Research Institute, Vibhavadee–Rangsit Highway, Laksi, Bangkok 10210, Thailand.; c Department of Physics, Faculty of Science, Thammasart University, Vibhavadee–Rangsit Highway, Bangkok, Thailand.

## Abstract

In the title mol­ecule, C_17_H_10_BrNO_3_, the mean planes of tricycle and bromo­phenyl fragments form a dihedral angle of 75.5 (1)°. In the crystal, π–π inter­actions [centroid–centroid distances = 3.556 (2) and 3.898 (8) Å] between the isoquinoline systems link mol­ecules into stacks parallel to the *a* axis. The crystal packing also exibits weak inter­molecular C—H⋯O hydrogen bonds.

## Related literature

The title compound was been obtained during our work on the synthesis of oxoaporphine from isoquinoline for use as a substrate for coupling reactions to obtain an oxoaporphine product, see: Cuny (2004 ▶); Lafrance *et al.* (2004 ▶). For related structures, see: Orito *et al.* (2000 ▶).
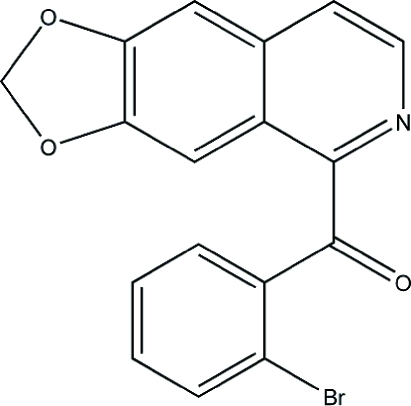


## Experimental

### 

#### Crystal data

C_17_H_10_BrNO_3_
*M* *_r_* = 356.17
Triclinic, 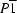

*a* = 7.6152 (6) Å
*b* = 7.8130 (6) Å
*c* = 12.0454 (9) Å
α = 98.339 (2)°
β = 94.982 (1)°
γ = 100.264 (2)°
*V* = 693.06 (9) Å^3^
*Z* = 2
Mo *K*α radiation
μ = 2.98 mm^−1^
*T* = 298 K
0.16 × 0.13 × 0.11 mm

#### Data collection

Bruker SMART CCD area-detector diffractometer
3157 measured reflections
2742 independent reflections
2276 reflections with *I* > 2σ(*I*)
*R*_int_ = 0.015

#### Refinement

*R*[*F* ^2^ > 2σ(*F* ^2^)] = 0.044
*wR*(*F* ^2^) = 0.130
*S* = 1.04
2742 reflections
199 parameters
H-atom parameters constrained
Δρ_max_ = 0.64 e Å^−3^
Δρ_min_ = −0.76 e Å^−3^

### 

Data collection: *SMART* (Bruker, 2007 ▶); cell refinement: *SAINT* (Bruker, 2007 ▶); data reduction: *SAINT*; program(s) used to solve structure: *SHELXS97* (Sheldrick, 2008 ▶); program(s) used to refine structure: *SHELXL97* (Sheldrick, 2008 ▶); molecular graphics: *XP* in *SHELXTL* (Sheldrick, 2008 ▶); software used to prepare material for publication: *SHELXL97*.

## Supplementary Material

Crystal structure: contains datablocks I, global. DOI: 10.1107/S1600536810051329/cv5003sup1.cif

Structure factors: contains datablocks I. DOI: 10.1107/S1600536810051329/cv5003Isup2.hkl

Additional supplementary materials:  crystallographic information; 3D view; checkCIF report

## Figures and Tables

**Table 1:** Hydrogen-bond geometry (Å, °)

*D*—H⋯*A*	*D*—H	H⋯*A*	*D*⋯*A*	*D*—H⋯*A*
C9—H9*A*⋯O3^i^	0.93	2.58	3.239 (3)	128

Symmetry code: (i) 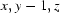.

**Fig. 1.**
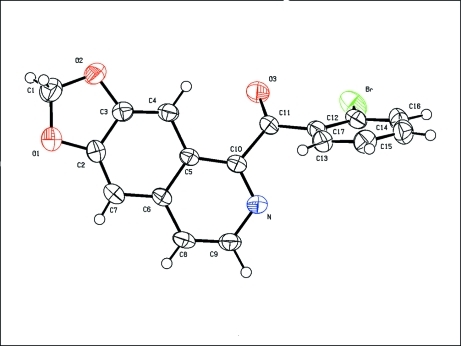
The molecular structure of (I) showing the atomic numbering and 50% probabilty displacement ellipsoids.

## supplementary crystallographic information

### Comment

The title compound (I) has been obtained in the framework of our work directed to the synthesis of oxoaporphine from isoquinoline to use it as a substrate for coupling reaction to reach an oxoaporphine product (Cuny *et al.*, 2004; Lafrance *et al.*, 2004).

In (I) (Fig. 1), all bond lengths and angles are normal and comparable with those observed in related compounds (Orito *et al.*, 2000). The C11—O3 bond length is 1.210 (3) Å - typical for carbonyl group. The mean planes of tricycle and bromophenyl fragments form a dihedral angle of 75.5 (1)°. The C5-C10-C11-C12 torsion angle is 146.1 (3)°.

In the crystal structure, weak intermolecular π-π interactions between the isoquinoline systems (Table 1) link the molecules into stacks parallel to the axis *a*. The crystal packing exibits also weak intermolecular C—H···O hydrogen bonds (Table 2).

### Experimental

The title compound was synthesized from piperonal in five steps. The 3,4-methylenedioxypiperonal was converted into phenylethylamine by the reaction with CH_3_NO_2_, NH_4_OAc and acetic acid at 90°C for 2 h.The phenylethylamine was then treated with 4-nitrobenzenesulfonyl chloride at room temperature for 40 h yielding sulfonamide. The sulfonamide further reacted with glyoxal compound at room temperature for 46 h. The resulting product, tetrahydroisoquinoline, was dehydrogenated under basic condition giving the title compound as pale yellow needles.

### Refinement

All H atoms were geometrically positioned (C—H 0.93-0.97 Å), and refined as riding, with *U*_iso_(H) = 1.2-1.5 *U*_eq_ of the parent atom.

### Crystal data

**Table:**

C_17_H_10_BrNO_3_	*Z* = 2
*M**_r_* = 356.17	*F*(000) = 356
Triclinic, *P*1	*D*_x_ = 1.707 Mg m^−^^3^
*a* = 7.6152 (6) Å	Mo *K*α radiation, λ = 0.71073 Å
*b* = 7.8130 (6) Å	Cell parameters from 25 reflections
*c* = 12.0454 (9) Å	θ = 25–35°
α = 98.339 (2)°	µ = 2.98 mm^−^^1^
β = 94.982 (1)°	*T* = 298 K
γ = 100.264 (2)°	Needle, colourless
*V* = 693.06 (9) Å^3^	0.16 × 0.13 × 0.11 mm

### Data collection

**Table:**

Bruker SMART CCD area-detector diffractometer	2276 reflections with *I* > 2σ(*I*)
Radiation source: fine-focus sealed tube	*R*_int_ = 0.015
graphite	θ_max_ = 30.5°, θ_min_ = 2.7°
phi and ω scans	*h* = −10→7
3157 measured reflections	*k* = −11→9
2742 independent reflections	*l* = −16→14

### Refinement

**Table:**

Refinement on *F*^2^	Primary atom site location: structure-invariant direct methods
Least-squares matrix: full	Secondary atom site location: difference Fourier map
*R*[*F*^2^ > 2σ(*F*^2^)] = 0.044	Hydrogen site location: inferred from neighbouring sites
*wR*(*F*^2^) = 0.130	H-atom parameters constrained
*S* = 1.04	*w* = 1/[σ^2^(*F*_o_^2^) + (0.0873*P*)^2^ + 0.147*P*] where *P* = (*F*_o_^2^ + 2*F*_c_^2^)/3
2742 reflections	(Δ/σ)_max_ = 0.001
199 parameters	Δρ_max_ = 0.64 e Å^−^^3^
0 restraints	Δρ_min_ = −0.76 e Å^−^^3^

### Special details

**Table:**

Geometry. All e.s.d.'s (except the e.s.d. in the dihedral angle between two l.s. planes) are estimated using the full covariance matrix. The cell e.s.d.'s are taken into account individually in the estimation of e.s.d.'s in distances, angles and torsion angles; correlations between e.s.d.'s in cell parameters are only used when they are defined by crystal symmetry. An approximate (isotropic) treatment of cell e.s.d.'s is used for estimating e.s.d.'s involving l.s. planes.
Refinement. Refinement of *F*^2^ against ALL reflections. The weighted *R*-factor *wR* and goodness of fit *S* are based on *F*^2^, conventional *R*-factors *R* are based on *F*, with *F* set to zero for negative *F*^2^. The threshold expression of *F*^2^ > σ(*F*^2^) is used only for calculating *R*-factors(gt) *etc*. and is not relevant to the choice of reflections for refinement. *R*-factors based on *F*^2^ are statistically about twice as large as those based on *F*, and *R*- factors based on ALL data will be even larger.

### Fractional atomic coordinates and isotropic or equivalent isotropic displacement parameters (Å^2^)

**Table:**

	*x*	*y*	*z*	*U*_iso_*/*U*_eq_	
Br	0.29325 (5)	0.84480 (4)	0.65647 (3)	0.05585 (17)	
O1	1.0229 (3)	0.6977 (3)	1.29657 (19)	0.0484 (6)	
O2	1.0035 (3)	0.9486 (3)	1.2189 (2)	0.0489 (6)	
O3	0.5942 (4)	0.8829 (3)	0.8581 (2)	0.0533 (6)	
N	0.5871 (4)	0.4413 (3)	0.8070 (2)	0.0372 (5)	
C1	1.0794 (5)	0.8853 (4)	1.3122 (3)	0.0541 (9)	
H1A	1.0405	0.9389	1.3813	0.065*	
H1B	1.2094	0.9160	1.3184	0.065*	
C2	0.9304 (4)	0.6499 (4)	1.1915 (2)	0.0352 (6)	
C3	0.9177 (4)	0.8020 (3)	1.1432 (2)	0.0353 (6)	
C4	0.8317 (4)	0.7975 (3)	1.0398 (2)	0.0358 (6)	
H4A	0.8249	0.9001	1.0104	0.043*	
C5	0.7512 (4)	0.6262 (3)	0.9773 (2)	0.0297 (5)	
C6	0.7642 (4)	0.4726 (3)	1.0269 (2)	0.0307 (5)	
C7	0.8554 (4)	0.4857 (3)	1.1363 (3)	0.0369 (6)	
H7A	0.8636	0.3861	1.1689	0.044*	
C8	0.6816 (4)	0.3074 (3)	0.9630 (2)	0.0375 (6)	
H8A	0.6847	0.2048	0.9932	0.045*	
C9	0.5975 (4)	0.2978 (3)	0.8574 (3)	0.0392 (6)	
H9A	0.5441	0.1871	0.8174	0.047*	
C10	0.6596 (4)	0.5985 (3)	0.8670 (2)	0.0322 (5)	
C11	0.6251 (4)	0.7482 (3)	0.8073 (2)	0.0353 (6)	
C12	0.6279 (4)	0.7207 (3)	0.6816 (2)	0.0330 (6)	
C13	0.7726 (4)	0.6592 (4)	0.6371 (3)	0.0411 (7)	
H13A	0.8574	0.6248	0.6850	0.049*	
C14	0.7927 (5)	0.6483 (4)	0.5236 (3)	0.0461 (7)	
H14A	0.8929	0.6123	0.4961	0.055*	
C15	0.6625 (5)	0.6914 (4)	0.4513 (3)	0.0491 (8)	
H15A	0.6734	0.6807	0.3744	0.059*	
C16	0.5171 (5)	0.7501 (4)	0.4921 (3)	0.0482 (8)	
H16A	0.4299	0.7786	0.4429	0.058*	
C17	0.5004 (4)	0.7668 (3)	0.6066 (3)	0.0372 (6)	

### Atomic displacement parameters (Å^2^)

**Table:**

	*U*^11^	*U*^22^	*U*^33^	*U*^12^	*U*^13^	*U*^23^
Br	0.0385 (2)	0.0639 (2)	0.0752 (3)	0.02005 (16)	0.00774 (18)	0.03108 (17)
O1	0.0518 (15)	0.0501 (11)	0.0419 (13)	0.0091 (10)	−0.0035 (10)	0.0090 (8)
O2	0.0525 (14)	0.0400 (10)	0.0478 (13)	0.0053 (9)	−0.0099 (10)	0.0000 (8)
O3	0.080 (2)	0.0352 (10)	0.0486 (13)	0.0286 (11)	0.0000 (11)	0.0041 (8)
N	0.0442 (14)	0.0287 (10)	0.0383 (13)	0.0081 (9)	0.0022 (10)	0.0042 (8)
C1	0.055 (2)	0.0509 (17)	0.052 (2)	0.0093 (15)	−0.0082 (15)	0.0023 (13)
C2	0.0324 (14)	0.0433 (13)	0.0326 (14)	0.0107 (11)	0.0065 (11)	0.0095 (10)
C3	0.0314 (14)	0.0341 (11)	0.0396 (15)	0.0059 (10)	0.0034 (11)	0.0043 (9)
C4	0.0365 (15)	0.0280 (10)	0.0431 (16)	0.0063 (10)	0.0019 (11)	0.0082 (9)
C5	0.0312 (13)	0.0267 (10)	0.0336 (13)	0.0083 (9)	0.0074 (10)	0.0076 (8)
C6	0.0318 (14)	0.0287 (10)	0.0347 (14)	0.0072 (9)	0.0089 (10)	0.0109 (8)
C7	0.0375 (16)	0.0368 (12)	0.0411 (16)	0.0098 (11)	0.0083 (12)	0.0166 (10)
C8	0.0454 (17)	0.0241 (10)	0.0459 (16)	0.0075 (10)	0.0103 (12)	0.0115 (9)
C9	0.0477 (18)	0.0257 (10)	0.0436 (16)	0.0058 (10)	0.0067 (12)	0.0048 (9)
C10	0.0348 (14)	0.0271 (10)	0.0367 (14)	0.0087 (9)	0.0049 (10)	0.0079 (9)
C11	0.0416 (16)	0.0304 (11)	0.0356 (15)	0.0103 (10)	0.0008 (11)	0.0092 (9)
C12	0.0339 (14)	0.0273 (10)	0.0379 (14)	0.0055 (9)	−0.0004 (11)	0.0095 (8)
C13	0.0434 (18)	0.0409 (13)	0.0393 (16)	0.0088 (12)	0.0000 (12)	0.0092 (10)
C14	0.0449 (19)	0.0476 (15)	0.0464 (19)	0.0103 (13)	0.0082 (14)	0.0064 (12)
C15	0.054 (2)	0.0549 (17)	0.0370 (17)	0.0032 (15)	0.0047 (14)	0.0136 (12)
C16	0.0415 (18)	0.0573 (17)	0.0460 (19)	0.0030 (14)	−0.0057 (14)	0.0229 (13)
C17	0.0309 (14)	0.0344 (12)	0.0473 (17)	0.0035 (10)	0.0005 (11)	0.0166 (10)

### Geometric parameters (Å, °)

**Table:**

Br—C17	1.903 (3)	C6—C7	1.417 (4)
O1—C2	1.361 (4)	C7—H7A	0.9300
O1—C1	1.432 (4)	C8—C9	1.360 (4)
O2—C3	1.378 (3)	C8—H8A	0.9300
O2—C1	1.411 (4)	C9—H9A	0.9300
O3—C11	1.210 (3)	C10—C11	1.509 (3)
N—C10	1.330 (3)	C11—C12	1.501 (4)
N—C9	1.361 (3)	C12—C13	1.396 (5)
C1—H1A	0.9700	C12—C17	1.398 (4)
C1—H1B	0.9700	C13—C14	1.381 (5)
C2—C7	1.356 (4)	C13—H13A	0.9300
C2—C3	1.411 (4)	C14—C15	1.382 (5)
C3—C4	1.349 (4)	C14—H14A	0.9300
C4—C5	1.438 (3)	C15—C16	1.374 (6)
C4—H4A	0.9300	C15—H15A	0.9300
C5—C10	1.414 (4)	C16—C17	1.385 (5)
C5—C6	1.431 (3)	C16—H16A	0.9300
C6—C8	1.412 (3)		
			
Cg1···Cg1^i^	3.556 (2)	Cg1···Cg2^ii^	3.898 (8)
			
C2—O1—C1	106.0 (2)	C6—C8—H8A	119.9
C3—O2—C1	106.2 (2)	C8—C9—N	123.6 (2)
C10—N—C9	117.2 (2)	C8—C9—H9A	118.2
O2—C1—O1	108.9 (2)	N—C9—H9A	118.2
O2—C1—H1A	109.9	N—C10—C5	124.7 (2)
O1—C1—H1A	109.9	N—C10—C11	112.6 (2)
O2—C1—H1B	109.9	C5—C10—C11	122.6 (2)
O1—C1—H1B	109.9	O3—C11—C12	121.7 (2)
H1A—C1—H1B	108.3	O3—C11—C10	121.5 (3)
C7—C2—O1	128.5 (3)	C12—C11—C10	116.8 (2)
C7—C2—C3	121.9 (3)	C13—C12—C17	117.7 (3)
O1—C2—C3	109.6 (2)	C13—C12—C11	118.7 (2)
C4—C3—O2	127.5 (3)	C17—C12—C11	123.4 (3)
C4—C3—C2	123.5 (2)	C14—C13—C12	121.5 (3)
O2—C3—C2	108.9 (2)	C14—C13—H13A	119.2
C3—C4—C5	116.7 (2)	C12—C13—H13A	119.2
C3—C4—H4A	121.6	C13—C14—C15	119.4 (3)
C5—C4—H4A	121.6	C13—C14—H14A	120.3
C10—C5—C6	116.8 (2)	C15—C14—H14A	120.3
C10—C5—C4	123.7 (2)	C16—C15—C14	120.4 (3)
C6—C5—C4	119.4 (2)	C16—C15—H15A	119.8
C8—C6—C7	121.2 (2)	C14—C15—H15A	119.8
C8—C6—C5	117.5 (2)	C15—C16—C17	120.0 (3)
C7—C6—C5	121.3 (2)	C15—C16—H16A	120.0
C2—C7—C6	117.1 (2)	C17—C16—H16A	120.0
C2—C7—H7A	121.5	C16—C17—C12	120.9 (3)
C6—C7—H7A	121.5	C16—C17—Br	117.6 (2)
C9—C8—C6	120.2 (2)	C12—C17—Br	121.5 (2)
C9—C8—H8A	119.9		
			
C3—O2—C1—O1	−5.7 (4)	C9—N—C10—C5	2.2 (5)
C2—O1—C1—O2	5.7 (4)	C9—N—C10—C11	−174.6 (3)
C1—O1—C2—C7	177.5 (3)	C6—C5—C10—N	−0.5 (5)
C1—O1—C2—C3	−3.5 (4)	C4—C5—C10—N	178.7 (3)
C1—O2—C3—C4	−177.3 (3)	C6—C5—C10—C11	175.9 (3)
C1—O2—C3—C2	3.5 (4)	C4—C5—C10—C11	−4.9 (5)
C7—C2—C3—C4	−0.1 (5)	N—C10—C11—O3	141.9 (3)
O1—C2—C3—C4	−179.2 (3)	C5—C10—C11—O3	−34.9 (5)
C7—C2—C3—O2	179.1 (3)	N—C10—C11—C12	−37.1 (4)
O1—C2—C3—O2	0.0 (4)	C5—C10—C11—C12	146.1 (3)
O2—C3—C4—C5	−179.5 (3)	O3—C11—C12—C13	131.7 (3)
C2—C3—C4—C5	−0.4 (5)	C10—C11—C12—C13	−49.3 (3)
C3—C4—C5—C10	−178.6 (3)	O3—C11—C12—C17	−42.9 (4)
C3—C4—C5—C6	0.5 (4)	C10—C11—C12—C17	136.1 (3)
C10—C5—C6—C8	−1.4 (4)	C17—C12—C13—C14	1.7 (4)
C4—C5—C6—C8	179.4 (3)	C11—C12—C13—C14	−173.3 (2)
C10—C5—C6—C7	179.1 (3)	C12—C13—C14—C15	−3.0 (4)
C4—C5—C6—C7	−0.2 (4)	C13—C14—C15—C16	2.1 (5)
O1—C2—C7—C6	179.4 (3)	C14—C15—C16—C17	0.2 (5)
C3—C2—C7—C6	0.5 (5)	C15—C16—C17—C12	−1.5 (4)
C8—C6—C7—C2	−179.9 (3)	C15—C16—C17—Br	−178.9 (2)
C5—C6—C7—C2	−0.3 (4)	C13—C12—C17—C16	0.6 (4)
C7—C6—C8—C9	−178.9 (3)	C11—C12—C17—C16	175.3 (2)
C5—C6—C8—C9	1.6 (5)	C13—C12—C17—Br	177.88 (19)
C6—C8—C9—N	0.1 (5)	C11—C12—C17—Br	−7.4 (3)
C10—N—C9—C8	−2.0 (5)		

Symmetry codes: (i) −*x*+1, −*y*+1, −*z*; (ii) −*x*, −*y*+1, −*z*.

### Hydrogen-bond geometry (Å, °)

**Table:**

*D*—H···*A*	*D*—H	H···*A*	*D*···*A*	*D*—H···*A*
C9—H9A···O3^iii^	0.93	2.58	3.239 (3)	128

Symmetry codes: (iii) *x*, *y*−1, *z*.
